# Specific deletion of protein phosphatase 6 catalytic subunit in Sertoli cells leads to disruption of spermatogenesis

**DOI:** 10.1038/s41419-021-04172-y

**Published:** 2021-09-27

**Authors:** Wen-Long Lei, Yuan-Yuan Li, Tie-Gang Meng, Yan Ning, Si-Min Sun, Chun-Hui Zhang, Yaoting Gui, Zhen-Bo Wang, Wei-Ping Qian, Qing-Yuan Sun

**Affiliations:** 1 Department of Reproductive Medicine, Peking University Shenzhen Hospital, Shenzhen, 518036 China; 2 CAS Key Laboratory of Quantitative Engineering Biology, Shenzhen Institute of Synthetic Biology, Shenzhen Institutes of Advanced Technology, Chinese Academy of Sciences, Shenzhen, 518055 China; 3 Guangdong and Shenzhen Key Laboratory of Male Reproductive Medicine and Genetics, Institute of Urology, Peking University Shenzhen Hospital, Shenzhen PKU-HKUST Medical Center, Shenzhen, 518036 China; 4 State Key Laboratory of Stem Cell and Reproductive Biology, Institute of Zoology, Chinese Academy of Sciences, Beijing, 100101 China; 5 Fertility Preservation Lab, Guangdong-Hong Kong Metabolism & Reproduction Joint Laboratory, Reproductive Medicine Center, Guangdong Second Provincial General Hospital, Guangzhou, 510317 China

**Keywords:** Spermatogenesis, Infertility

## Abstract

Protein phosphatase 6 (PP6) is a member of the PP2A-like subfamily, which plays significant roles in numerous fundamental biological activities. We found that PPP6C plays important roles in male germ cells recently. Spermatogenesis is supported by the Sertoli cells in the seminiferous epithelium. In this study, we crossed *Ppp6c*^*F/F*^ mice with *AMH-Cre* mice to gain mutant mice with specific depletion of the *Ppp6c* gene in the Sertoli cells. We discovered that the PPP6C cKO male mice were absolutely infertile and germ cells were largely lost during spermatogenesis. By combing phosphoproteome with bioinformatics analysis, we showed that the phosphorylation status of β-catenin at S552 (a marker of adherens junctions) was significantly upregulated in mutant mice. Abnormal β-catenin accumulation resulted in impaired testicular junction integrity, thus led to abnormal structure and functions of BTB. Taken together, our study reveals a novel function for PPP6C in male germ cell survival and differentiation by regulating the cell-cell communication through dephosphorylating β-catenin at S552.

## Introduction

Spermatogenesis is an intricate developmental process by which spermatogonial stem cells (SSCs) renovate and differentiate to produce mature spermatozoa, consisting of three phases: spermatogonial mitosis, spermatocytic meiosis, and spermiogenesis [1]. Any errors in this process can result in serious outcomes including infertility [2, 3]. In addition to germ cells, there are some other somatic cells in the seminiferous epithelium, such as Sertoli cells, which have significant functions in spermatogenesis. Germ cells and Sertoli cells form a series of connection structures to support the transfer of signaling elements, attachment, and germ cell differentiation [4]. Destruction of Sertoli cell can disrupt germ cell differentiation and spermatogenesis.

In the mammalian testis, blood‑testis barrier (BTB) is found between the adjacent Sertoli cells within the seminiferous tubules, which divided the seminiferous epithelium into the basal and the apical compartments. Germ cell meiosis completion, spermiogenesis occur in the apical compartment, whereas SSCs division and differentiation to preleptotene spermatocytes take place in the basal compartment [5, 6]. Therefore, the BTB forms an immunological microenvironment for meiotic and postmeiotic cells [7]. Unlike other blood-tissue barriers forming by a tight junction (TJ) [8], the BTB is constituted by several types of cell-cell junctions, such as TJ, adhesion junction (AJ), gap junction (GJ) and desmosome-like junction, and numerous junctional proteins are involved in the formation of BTB [7].

Protein phosphorylation and dephosphorylation always occur in spermatogenesis [9]. The dynamic changes of protein phosphorylation levels are controlled by a conserved series of protein kinases and protein phosphatases. Among these numerous phosphatases, PP2A, PP4, and PP6 constitute the type 2 A subfamily within the serine/threonine protein phosphatase family [10]. Type 2 A subfamily have important functions in lots of fundamental cellular processes [11, 12]. Like other type 2 A phosphatases, PP6 also works as a holoenzyme and is evolutionally conserved among eukaryotes, indicating its importance. Previous studies found that PP6 plays pivotal roles in cell/organ size regulation, inflammatory signaling, pre-mRNA splicing [13–15], the G1-S transition, S phase arrest [16–18], and mitotic spindle formation [19]. Moreover, a study showed that PP6 may have an effect on the dephosphorylation of γ-H2AX [20]. Our lab found that conditional knockout (cKO) of PPP6C in male germ cells leads to complete infertility and germ cells are arrested at the pachytene stage [21]. PPP6C is also occurs in Sertoli cells, however, the functions of PPP6C in Sertoli cells is still absolutely unknown.

In this study, we crossed *Ppp6c*^*F/F*^ mice with *AMH-Cre* mice to gain mutant mice with specific depletion of the *Ppp6c* gene in Sertoli cells. We discovered that the PPP6C-deficient cKO mice were absolutely infertile and germ cells were evidently lost during spermatogenesis. By combing phosphoproteome with bioinformatics analysis, we showed that the phosphorylation status of β-catenin at S552 was significantly upregulated in cKO group. β-catenin abnormal accumulation resulted in impaired testicular junction integrity, thus led to the abnormal structures and functions of BTB. Thus, our work for the first time reveals a novel role of PPP6C in determining germ cell death and differentiation by regulating the cell-cell communication through dephosphorylating β-catenin at S552.

## Results

### Specific deletion of *Ppp6c* gene by *AMH-Cre* results in male infertility

**Fig. 1:**
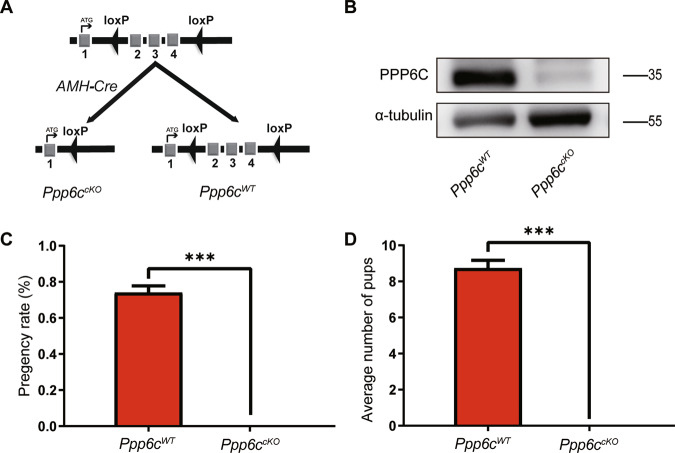
PPP6C is essential for male fertility. **A** Schematic diagram of deletion of *Ppp6c* exons and creation of *Ppp6c Δ* allele by *AMH-Cre*-mediated recombination in Sertoli cells. **B** Western blotting analysis of PPP6C protein in *Ppp6c*^*WT*^ and *Ppp6c*^*cKO*^ Sertoli cells of 8-week-old mice. α-tubulin was detected as an internal control. **C** Pregnancy rates (%) of plugged wild-type females after mating with *Ppp6c*^*cKO*^ or *Ppp6c*^*WT*^ 8-week-old males. **D** Average litter size of plugged wild-type females after mating with *Ppp6c*^*cKO*^ or *Ppp6c*^*WT*^ 8-week-old males. For this part, at least 3 mice (8-week-old) of each genotype were used for analysis. Data are presented as the mean ± SEM. *P* < 0.05(*), 0.01(**), or 0.001(***).

We gained mice in which the *Ppp6c* gene was specifically deleted in Sertoli cells to study the PPP6C functions in Sertoli cells. We used *Ppp6c*^*F/F*^ mice in which exons II-IV of the *Ppp6c* gene were flanked with Loxp sites [22]. PPP6C was disrupted in Sertoli cells by crossing *Ppp6c*^*F/F*^ mice with *AMH-Cre* transgenic mice (referred to as *Ppp6c*^*cKO*^) (Fig. 1A). *AMH-Cre* recombinase had recombinase activities in Sertoli cells [23]. PPP6C-deletion efficiency in Sertoli cells was analyzed by testing the protein levels in Sertoli cells. The results (Fig. 1B) indicated that PPP6C was absent in Sertoli cells of *Ppp6c*^*cKO*^ mice. So, we gained Sertoli cell-specific knockout mice for PPP6C. The breeding assays indicated that the *Ppp6c* cKO mice were infertile (Fig. 1C, D).

### *Ppp6c* depletion causes abnormal spermatogenesis

**Fig. 2:**
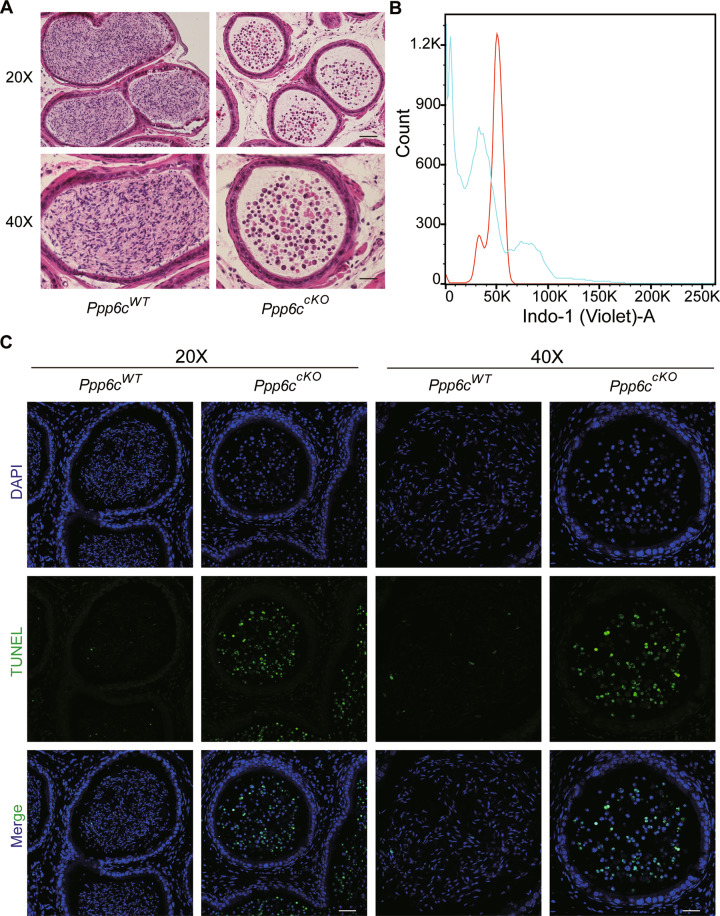
PPP6C is required for spermatogenesis. **A** Histological analysis of the caudal epididymides of the *Ppp6c*^*WT*^ and *Ppp6c*^*cKO*^ mice. Scale bar: (top) 100 μm; (bottom) 50 μm. **B** DNA content analysis of control and cKO cells derived epididymides by FACS. The red peak represents the control, which had normal haploid DNA contents; while the blue peak represents the *Ppp6c*^*cKO*^ cells derived epididymides, which were abnormal. **C** TUNEL immunofluorescence staining of the epididymides of *Ppp6c*^*WT*^ and *Ppp6c*^*cKO*^. Scale bar: (left) 50 μm, (right) 20 μm. Green: TUNEL positive signal; Blue: DAPI. At least 3 mice (8-week-old) of each genotype were used for analysis.

**Fig. 3:**
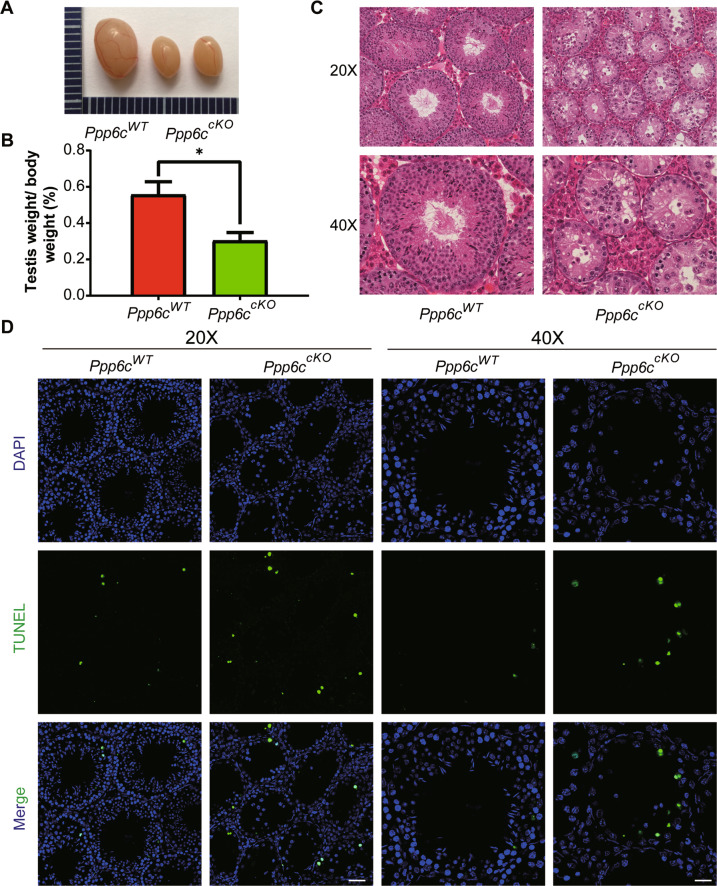
PPP6C knockout in mouse Sertoli cells by *AMH-Cre* results in testicular atrophy and the apoptosis of germ cells. **A** The testes of *Ppp6c*^*cKO*^ were smaller than those of the *Ppp6c*^*WT*^ (8-week-old, the same as below). **B** Testis weight to body weight ratio of *Ppp6c*^*WT*^ and *Ppp6c*^*cKO*^ mice (*n* = 3). Data are presented as the mean ± SEM. *P* < 0.05(*), 0.01(**) or 0.001(***). **C** Histological analysis of the seminiferous tubules of the *Ppp6c*^*WT*^ and *Ppp6c*^*cKO*^ mice. Scale bar: (top) 100 μm; (bottom) 50 μm. **D** TUNEL immunofluorescence staining of the testes of *Ppp6c*^*WT*^ and *Ppp6c*^*cKO*^. Scale bar: (left) 50 μm, (right) 20 μm. Green: TUNEL positive signal; Blue: DAPI. At least 3 mice (8-week-old) of each genotype were used for analysis.

To determine the causes of infertility in *Ppp6c*^*cKO*^ mice, we firstly analyzed the histology of the epididymides by hematoxylin and eosin (H&E) staining. The results showed that circular-cellular debris rather than mature spermatozoa were commonly found in the epididymal lumens of *Ppp6c*^*cKO*^ mice (Fig. 2A). In particular, large numbers of what appear to be highly vacuolated round cells were observed in the epididymides of *Ppp6c*^*cKO*^ males. And these circular cells diminished with age (Fig. S1A). Then, we performed immunofluorescence staining of the DAPI to characterize the shape of sperm in epididymal lumens. The results revealed that the shape of sperm in *Ppp6c*^*WT*^ was hook-like, but the shape of sperm in *Ppp6c*^*cKO*^ was round (Fig. S2A), suggesting that the process of spermatogenesis was affected in *Ppp6c*^*cKO*^ mice. Also, we found that the majority of *Ppp6c*^*cKO*^ germ cells in epididymal lumens were not typical haploid (Fig. 2B). We speculated that germ cells were undergoing apoptosis. So, we performed TUNEL assay and found that germ cells underwent apoptosis in the *Ppp6c*^*cKO*^ mice (Fig. 2C). Then we found the testes of *Ppp6c*^*cKO*^ mice were much smaller than controls (Fig. 3A) and the testis weight to body weight ratio of *Ppp6c*^*cKO*^ was lower (Fig. 3B). And the variations of testes were more drastic with age (Fig. S3A, B). Also, compared with controls, the number of germ cells were sharply reduced, and there are nearly no round and elongated spermatids in *Ppp6c*^*cKO*^ mice (Fig. 3C). And the variations of histomorphology were more distinct with age (Fig. S3C). Similarly, we performed TUNEL assay to detect apoptosis. The results showed that germ cells underwent apoptosis in the *Ppp6c*^*cKO*^ mice (Fig. 3D).

**Fig. 4:**
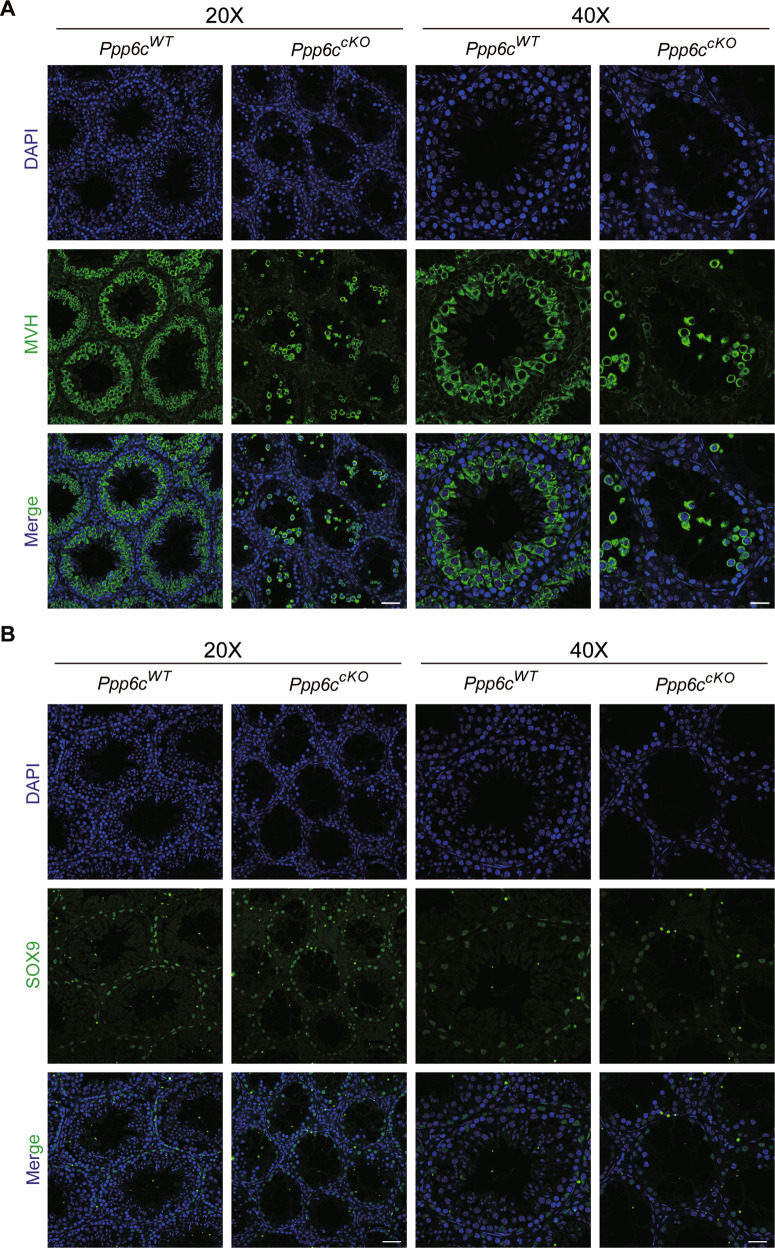
PPP6C knockout in mouse Sertoli cells by *AMH-Cre* results in the loss of germ cells. **A** MVH (green) immunofluorescence analysis of the *Ppp6c*^*WT*^ and *Ppp6c*^*cKO*^ mice showed that the amount of the germ cells were decreased Scale bar: (left) 50 μm, (right) 20 μm. (**B**) SOX9 (green) immunofluorescence analysis of the *Ppp6c*^*WT*^ and *Ppp6c*^*cKO*^ mice showed that the amount and location of the Sertoli cells were similar. Scale bar: (left) 50 μm, (right) 20 μm. Nuclei are stained with DAPI. At least 3 mice of each genotype were used for analysis.

According to the above results, we discovered that *Ppp6c* depletion causes impaired spermatogenesis and the number of germ cells were reduced. To confirm the results, we performed immunofluorescence by using the germ cell marker MVH. Immunofluorescence results indicated that the number of MVH positive signals was decreased in *Ppp6c*^*cKO*^ testicular sections compared with those in *Ppp6c*^*WT*^ (Fig. 4A). Then we wanted to know whether it influenced the Sertoli cells in PPP6C null males. We performed immunofluorescence by using the Sertoli cells marker SOX9. The results showed that the number and location of Sertoli cells did not show the obvious change (Fig. 4B).

**Fig. 5:**
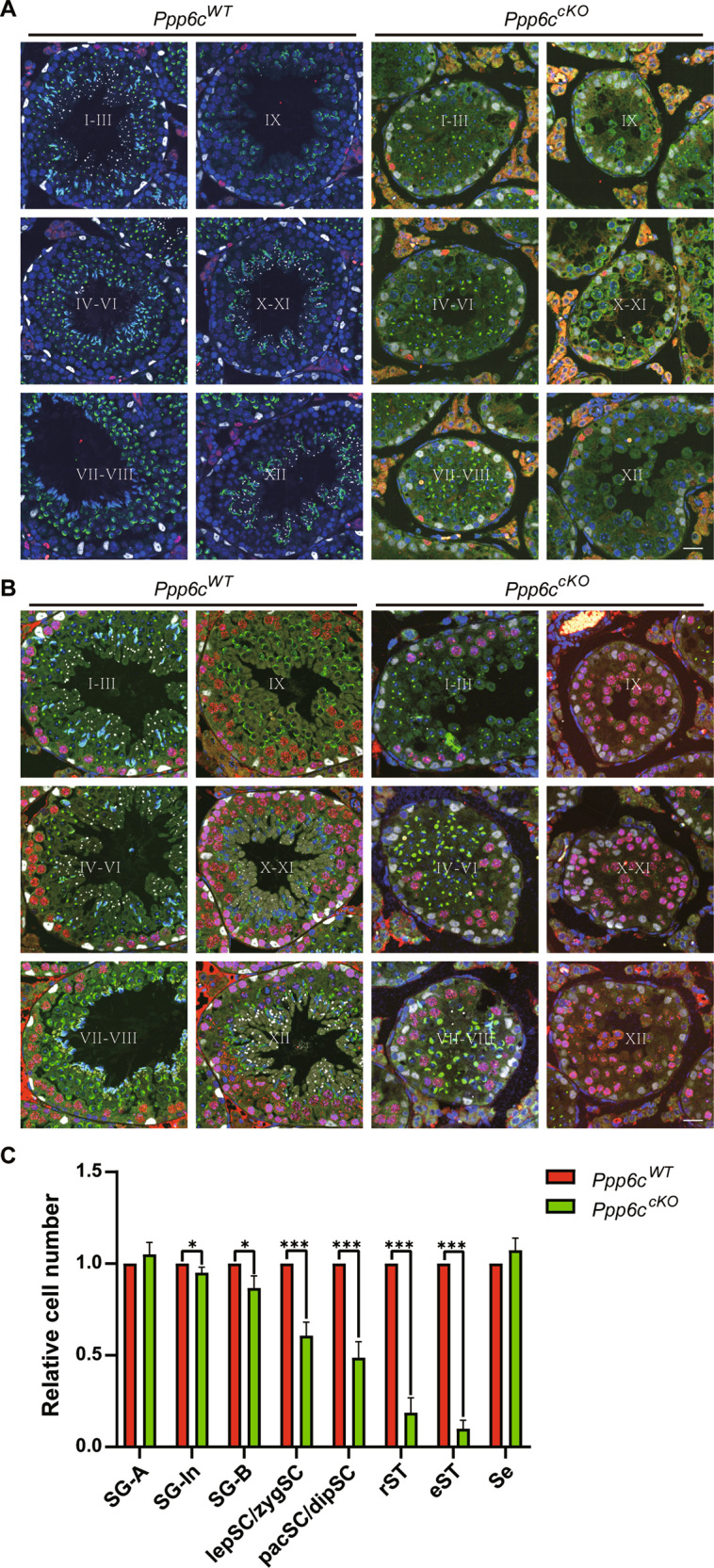
*Ppp6c* cKO mice arrest spermatogenesis at step 7-8 spermatids. **A** Determination of stages (I–XII) and identification of cell types using a combination of IHC and PNA-lectin histochemistry. Three-μm-thick sections were double-immunostained for PLZF (red) and SOX9 (white), then stained with PNA-lectin histochemistry (green) and DAPI (blue). Scale bar: 10 μm. **B** Determination of stages (I–XII) and identification of cell types using a combination of IHC and PNA-lectin histochemistry. Three-μm-thick sections were double-immunostained for SYCP3 (red) and SOX9 (white), then stained with PNA-lectin histochemistry (green) and DAPI (blue). Scale bar: 10 μm. **C** Quantification of spermatogenic cell types in *Ppp6c*^*WT*^ and *Ppp6c*^*cKO*^ mice based on the images shown in A-B. For each cell type, at least 150 tubules from three mice were counted. The average numbers of cells per tubule were converted to ratios and compared between *Ppp6c*^*WT*^ and *Ppp6c*^*cKO*^ mice. SG-A, type A spermatogonia; SG-In, type In spermatogonia; SG-B, type B spermatogonia; lepSC/zygSC leptotene spermatocytes/zygotene spermatocytes, pacSC/dipSC pachytene spermatocytes/diplotene spermatocytes, rST round spermatids, eST elongating spermatids, Se Sertoli cells.

As the spermatogenesis was disturbed in *Ppp6c*^*cKO*^ mice, we hope to determine which stages of spermatogenesis were affected in PPP6C cKO mice. Spermatogenesis can be subdivided into 12 stages and 16 steps in mouse testes by combining PNA lectin with DAPI [24, 25]. Also, we could determine different cell types in each phase by using IHC for PLZF, SYCP3, and SOX9, markers for type A spermatogonia, spermatocytes, and Sertoli cells, respectively. We found that spermatogenesis of PPP6C-deficient mice was blocked at stages VII-VIII (step 7-8 spermatids) (Fig. 5A, B). Then we quantified the cell numbers in seminiferous tubules and found that the numbers of type In spermatogonia, type B spermatogonia, leptotene spermatocytes/zygotene spermatocytes, pachytene spermatocytes/diplotene spermatocytes, round spermatids, as well as elongating spermatids were all reduced (Fig. 5C). These data showed that Sertoli cell-specific PPP6C knockout results in spermatogenesis failure and thus male infertility.

### A large-scale quantification of the phosphoproteome in PPP6C null Sertoli cells

**Fig. 6:**
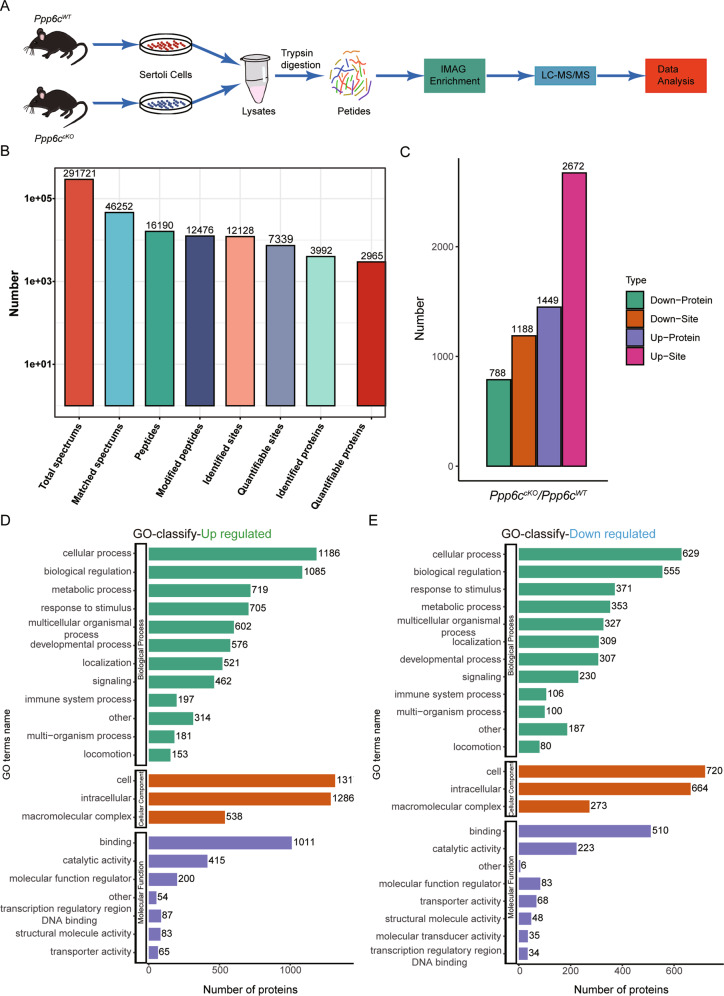
Global analysis of quantitative phosphoproteome. **A** Flowchart of the identification of the quantitative phosphoproteome in *Ppp6c*^*WT*^ and *Ppp6c*^*cKO*^ mice Sertoli cells. **B** The resulting MS/MS data were processed using MaxQuant search engine (v.1.6.6.0). A summary of the identified and quantified phosphoproteome and p-sites. **C** The number of up- and downregulated proteins and p-sites in this study. **D** GO analysis of the significantly upregulated proteins for biological process, molecular function, and KEGG pathway. **E** GO analysis of the significantly downregulated proteins for biological process, molecular function, and KEGG pathway.

To investigate a comprehensive perspective of the mechanisms of PPP6C depletion in Sertoli cells, we isolated them from *Ppp6c*^*WT*^ and *Ppp6c*^*cKO*^ testes at 5–7 dpp and systematically profiled the quantitative phosphoproteome (Fig. 6A). We applied a 4D label-free quantification approach by high-resolution liquid chromatography-mass spectrometry (LC-MS) [26]. In total, we identified 16,190 unique peptides and 12,476 of them were modified peptides. By mapping the phosphopeptides to their corresponding protein sequences, we comprehensively quantified 2965 phosphoproteins with 7339 unique phosphorylation sites (Fig. 6B). Among these quantified phosphoproteins and phosphorylation sites, we defined significantly different (*p* < 0.05 by Student’s *t* test) proteins and used a criterion of 1.5-fold change or greater between these two groups as differential protein candidates. Subsequently, 788 downregulated proteins and 1449 upregulated proteins were identified. Similarly, 1188 downregulated phosphorylation sites and 2672 upregulated phosphorylation sites in were identified (Fig. 6C). To obtain more comprehensive information, we then performed Gene Ontology (GO) annotation. These proteins were sorted by Gene Ontology annotation based on: biological process, cellular component, and molecular function. GO analysis of upregulated proteins showed that 576 proteins were related to the processes of development (Fig. 6D), and GO analysis of downregulated proteins showed that 307 proteins were related to the processes of development (Fig. 6E). Then, we performed these proteins to bioinformatics enrichment analysis with GO and KEGG databases. These analyses revealed that these differential proteins were closely involved in adherens junctions, tight junctions, cell junction, and microtubule (Fig. S4), suggesting PPP6C could be important in cell connection and multiple cellular signaling regulation. Together, the phosphoproteome in PPP6C null Sertoli cells analysis suggested multiple new roles of PPP6C in cellular physiology, especially in cell communication and cell connection.

### Testicular junction integrity is impaired in *Ppp6c*^*cko*^ testes

**Fig. 7:**
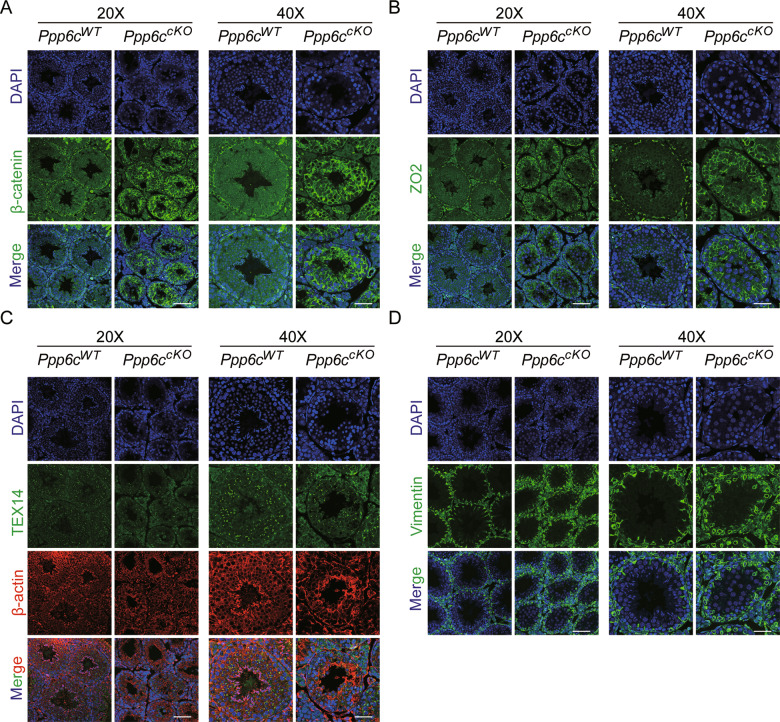
Disruption of the BTB integrity in *Ppp6c*^*cKO*^ mice. **A** β-Catenin (a marker of adherens junctions, green) immunofluorescence of *Ppp6c*^*WT*^ and *Ppp6c*^*cKO*^ mice testes. Scale bar: (left) 50 μm, (right) 20 μm. **B** ZO2 (a marker of tight junctions, green) immunofluorescence of *Ppp6c*^*WT*^ and *Ppp6c*^*cKO*^ mice testes. Scale bar: (left) 50 μm, (right) 20 μm. **C** TEX14 (a marker of a marker of testicular intercellular junctions, green) and β-actin (red) immunofluorescence of *Ppp6c*^*WT*^ and *Ppp6c*^*cKO*^ mice testes. Scale bar: (left) 50 μm, (right) 20 μm. **D** Vimentin (a marker for Sertoli apical extensions, green) immunofluorescence of *Ppp6c*^*WT*^ and *Ppp6c*^*cKO*^ mice testes. Scale bar: (left) 50 μm, (right) 20 μm. Nuclei are stained with DAPI.

According to the above-presented phosphoproteome data, we found that these differential phosphorylated proteins were closely related in cell junction and communication. Because of the loss of germ cells and the analyses of phosphoproteome data in *Ppp6c*^*cko*^ testes, we speculated that testicular junction integrity is impaired in PPP6C null mice. To verify the hypothesis, we firstly analyzed the expression of AJ (β-catenin) and TJ [zonula occludens 2 (ZO-2)] proteins in control and mutant testes. β-catenin was occured in the basal and lower adluminal compartment of control (Fig. 7A). Similar to β-catenin expression, ZO-2 was also occured in the basal compartment in WT testes (Fig. 7B). In contrast, mislocalization of β-catenin and ZO-2 was occured in *Ppp6c*^*cKO*^ mice (Fig. 7A, B). The testis-expressed gene 14 (TEX14), is mainly occurred in germ cell intercellular bridges and it is essential for spermatogenesis [27]. We found that mislocalization of TEX14 and β-actin was present in *Ppp6c*^*cKO*^ testes by using immunofluorescence (Fig. 7C), suggesting defects in testicular junctional complexes.

Sertoli cells also are polarized cells that extend from the basement membrane to the lumen of seminiferous tubules [28]. The apical extensions of Sertoli cells are contact with germ cells, directing their migration towards the lumen of the seminiferous tubules. We examined Sertoli cell apical extensions (marked by Vimentin) and observed the loss of apical extensions (Fig. 7D). These data showed that PPP6C-deletion influence the integrity of BTB, finally resulting in the loss of germ cells.

### PPP6C directly dephosphorylates β-catenin

**Fig. 8:**
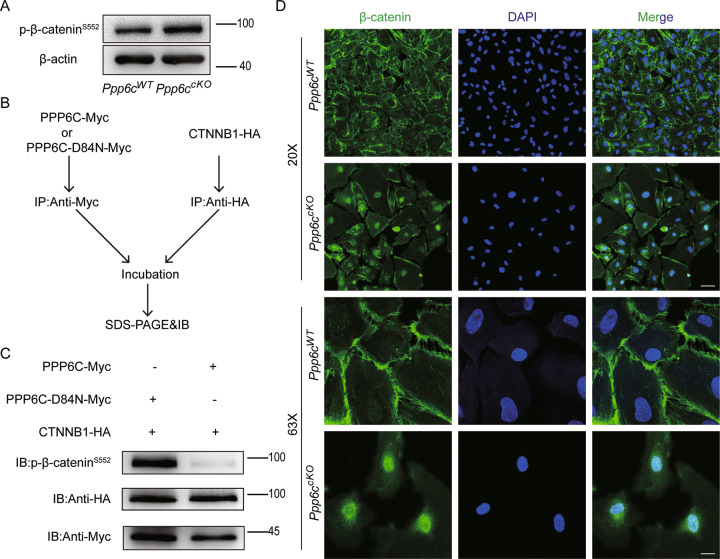
PPP6C directly dephosphorylates β-catenin. **A** Western blotting analysis of p-β-catenin^S552^ protein in *Ppp6c*^*WT*^ and *Ppp6c*^*cKO*^ Sertoli cells of mice. β-actin was detected as an internal control. At least 3 mice of each genotype were used for analysis. **B** A schematic diagram of in vitro phosphatase assays which were performed by using purified PPP6C (WT or D84N) and CTNNB1. **C** PPP6C (WT or D84N) was incubated with CTNNB1 to perform the in vitro phosphatase assays. Immunoblotting of the assay products exhibited an effective elimination of phospho-β-catenin (S552) by PPP6C, but not by the phosphatase-dead PPP6C (D84N). **D** β-Catenin immunofluorescence of *Ppp6c*^*WT*^ and *Ppp6c*^*cKO*^ Sertoli cells of mice. Scale bar: (top) 50 μm, (bottom) 10 μm.

To explore the mechanism underlying PPP6C-deletion-caused abnormal spermatogenesis, we analysed the detailed information on differential protein groups and p-sites (Supplementary Table 1) and found that the phosphorylation status of β-catenin at S552 (a marker of adherens junctions) was significantly upregulated in cKO group. By using immunoblotting, we tested the phosphorylation level of β-catenin at S552 and found that the phosphorylation status of β-catenin at S552 was significantly upregulated in cKO group (Fig. 8A), indicating that PPP6C could regulate the phosphorylation status of β-catenin. By using in vitro phosphatase assays, purified PPP6C (WT or D84N) was incubated with isolated CTNNB1 (Fig. 8B). As expected, we found an obvious elimination of phospho-β-catenin (S552) by PPP6C, but not by the phosphatase-dead PPP6C (D84N) (Fig. 8C). All observations thus suggested that PPP6C could directly dephosphorylate β-catenin. Generally, phosphorylation of β-catenin results in a weakening of the cadherin–β-catenin interaction, directing β-catenin into signaling mode. A study suggested that the phosphorylation of β-catenin at Ser552 was related with nuclear accumulation and transcriptional activation [29]. Therefore, we tested the locations of β-catenin in Sertoli cells from *Ppp6c*^*WT*^ and *Ppp6c*^*cKO*^ testes by immunofluorescence and found the nuclear accumulation of β-catenin in cKO mice (Fig. 8D). These data suggested that PPP6C deletion caused the abnormal nuclear accumulation of β-catenin and weakened of the cadherin–β-catenin interaction.

## Discussion

PP6 is a member of the PP2A-like subfamily and has a functional role in mitosis [19, 30]. Our lab previously reported that a conditional knockout of PPP6C in oocytes from growing follicles resulted in female subfertility, and a conditional knockout of PPP6C in oocytes from the primordial follicle stage, caused female infertility [22, 31]. As for male, we found that PPP6C is also critical for fertility and germ cell meiosis [21].

Sertoli cells play a very important role in spermatogenesis. In the seminiferous epithelium, cell-cell communications are hold by Sertoli-germ cell junctions and Sertoli-Sertoli cell junctions. There are some other junctions besides the tight junctions, such as the desmosome-like junction and the ectoplasmic specialization (ES) [32, 33]. At the early phase of the epithelial cycle, it is essential for the preleptotene spermatocytes to cross the BTB to enter the apical compartment and to get ready for meiotic progression by the disassembly and reassembly of the basal ES [32, 34]. During the procedures described above, BTB-related proteins, such as β-Catenin (a marker of adherens junctions), ZO2 (a marker of tight junctions), TEX14 (a marker of testicular intercellular junctions), and Vimentin (a marker for Sertoli apical extensions) play critical roles. In our study, we found that these proteins had abnormal expressions and locations in *Ppp6c*^*cKO*^ male mice. We speculated that the defects in testicular junctional complexes result in the abnormal structures and functions of BTB. Finally, PPP6C depletion causes male infertility and the loss of germ cells.

As one of the mostly characterized posttranslational modifications (PTMs), protein phosphorylation plays important roles in the adjustment of spermatogenesis. For the past few years, phosphoproteomic techniques have identified and quantified of thousands of phosphorylation sites in a single run [35, 36]. Although some large-scale studies have reported protein phosphorylation in different tissues and cells, the identification of critical regulatory protein phosphatase from the Sertoli cells of data has never been reported. To gain a comprehensive perspective of the mechanisms of PPP6C depletion in Sertoli cells, we isolated Sertoli cells from *Ppp6c*^*WT*^ and *Ppp6c*^*cKO*^ testes at 5–7 dpp and systematically profiled the quantitative phosphoproteome. The results showed that 788 downregulated proteins and 1449 upregulated proteins were identified. Similarly, 1188 downregulated phosphorylation sites and 2672 upregulated phosphorylation sites were identified. Then, we analysed and found that these differential proteins were closely involved in adherens junctions, tight junctions, cell junction, microtubule, suggesting PPP6C could be important in cell connection and multiple cellular signaling regulation. We also found that the phosphorylation status of β-catenin at S552 was significantly upregulated in cKO group, indicating PPP6C could regulate the phosphorylation status of β-catenin.

The multifunctional protein β-catenin has significant functions in both the canonical Wnt signaling pathway and intercellular adhesion. It is essential for lots of developmental processes [37, 38]. β-catenin is a member of the cadherin/catenin complexes. At adherens junctions, newly synthesized β-catenin is controled by E-cadherin, thereby controlling the actin cytoskeleton [39]. As for the canonical pathway, in the absence of Wnt, β-catenin can be degraded by the destruction complex, which is composed of the casein kinase 1 (CK1), scaffolding protein Axin, glycogen synthase kinase 3 (GSK3) and the tumor suppressor adenomatous polyposis coli gene product (APC). CK1α can phosphorylate β-catenin at serine 45 and GSK3 can phosphorylate β-catenin at threonine 41, serine 37, and serine 33 by GSK3. The E3 ubiquitin ligase β-Trcp can bind β-catenin at serine 33 and 37 when the two sites were phosphorylated, finally leading to β-catenin ubiquitination and degradation [40]. APC has a critical role in ensuring the ubiquitin conjugation of phosphorylated β-catenin. In the absence of APC, PP2A can dephosphorylated β-catenin [41]. This successive elimination of β-catenin protects β-catenin from entering the nucleus. The nuclear accumulation of β-catenin is a symbol of activated canonical Wnt/β-catenin signaling [42]. Active Wnt signaling destroys the destruction complex and induces the accumulation of β-catenin, which travels to the nucleus to interact with TCF/LEF and activates the transcriptional activity of Wnt target gene [43].

Generally, phosphorylation of β-catenin results in an instability of the β-catenin–cadherin complexs and direct β-catenin into signaling mode. A study suggested that the phosphorylation of β-catenin at Ser552 was related with nuclear accumulation and transcriptional activation [29]. Chang et al. reported that β-catenin was occurred in Sertoli cells especially in cell membrane. cKO of β-catenin in Sertoli cells by *AMH-Cre* caused no detectable abnormalities, whereas activation caused severe phenotypes, such as germ cell depletion and testicular cord disruption [44]. Other investigations found that activation of β-catenin in Sertoli cells by *Amhr2-Cre* resulted in male infertility and the loss of germ cells [45, 46]. In our present study, we found that the phenotypes in *Ppp6c*^*cKO*^ mice are similar to those of previously reported conditional activated allele of the β-catenin in Sertoli cells by using *AMH-Cre* or *Amhr2-Cre* by transgenic mouse, including male infertility and the loss of germ cells. We might provide new evidence to show that specific deletion of *Ppp6c* gene by *AMH-Cre* increases the phosphorylation status of β-catenin at S552. Phosphorylation of β-catenin results in an instability of the β-catenin–cadherin complexs and the integrity of BTB, directing β-catenin into signaling mode, finally resulting in the loss of germ cells and male infertility.

## Materials and methods

### Mice

Mice with disrupted *Ppp6c* in Sertoli cells (referred to as *Ppp6c*^*cKO*^) were created by crossing *Ppp6c*^*F/F*^ mice with *AMH*-*Cre* mice. The *Ppp6c*^*F/F*^ (referred to as *Ppp6c*^*WT*^) male mice were defined as control. Both these mouse lines have C57BL/6 J genomic background. Genotyping PCR for *Ppp6c* gene was experimented using the following primers: forward: GCAGAGGATGGGGTCACATAG, and reverse: ATCTCTGAACCAATTCTGGAG. The PCR conditions were as follows: 94 °C for 5 min; 35 rounds of 94 °C for 30 sec, 56 °C for 30 sec, and 72 °C for 30 sec; and 72 °C for 5 min. Genotyping PCR for *AMH*-*Cre* was experimented using the following primers: forward: TCCAATTTACTGACCGTACACCAA, and reverse: CCTGTACCTGGCAATTTCGGCTA. The PCR conditions were as follows: 94 °C for 5 min; 35 rounds of 94 °C for 30 sec, 62 °C for 30 sec, and 72 °C for 30 sec; and 72 °C for 5 min.

### Antibodies

PPP6C antibody (rabbit, A300-844A; Bethyl Laboratories, Inc.); SYCP3 antibody (rabbit, NB300-231; Novus Biologicals); α-tubulin antibody (rabbit, 2144; Cell Signaling Technology, Inc.); β-actin antibody (mouse, 3700; Cell Signaling Technology, Inc.); Phospho-β-Catenin (Ser552) (D8E11) antibody (rabbit, 5651; Cell Signaling Technology, Inc.); SYCP3 antibody (mouse, sc-74569; Santa Cruz); γH2AX antibody (rabbit, 9718; Cell Signaling Technology, Inc.); MVH antibody (mouse, ab27591; abcam); SYCP1 antibody (rabbit, ab15090; abcam); SOX9 antibody (rabbit, AB5535, Sigma-Aldrich); PLZF antibody (goat, AF2944, R&D Systems); β-catenin antibody (rabbit, 51067-1-AP, Proteintech); ZO2 antibody (rabbit, 18900-1-AP, Proteintech); TEX14 antibody (rabbit, 18351-1-AP, Proteintech); Vimentin antibody (rabbit, 10366-1-AP, Proteintech); HA-Tag mab (mouse, AE008; ABclonal); c-Myc antibody (mouse, m4439; sigma); green-fluorescent Alexa Fluor® 488 conjugate of lectin PNA (L21409, Thermo). Horseradish peroxidase–conjugated secondary antibodies were purchased from Zhongshan Golden Bridge Biotechnology Co, LTD (Beijing). Alexa Fluor 488–conjugated antibody and Alexa Fluor 594–conjugated antibody were purchased from Life Technologies.

### Breeding assay

Males (8–9 weeks) of different groups were individually performed for the experiment. Each male mouse was caged with two wild-type ICR strain females, then vaginal plugs were checked every morning. The number of pups in each cage was counted within a week of birth. Each male underwent at least six cycles of the above breeding assay.

### Immunoblotting

To prepare protein extracts, Sertoli cells isolated from testes were put into cold RIPA buffer supplemented with protease and phosphatase inhibitor cocktail (Roche Diagnostics). Then, the Sertoli cell lysates were incubated on ice for 20 min and centrifuged at 4 °C, 12000 rpm for 15 min. The supernatant was transferred to a new tube and equal volume 2x loading buffer was added. After being boiled at 95 °C for 10 min, the lysates were used for immunoblotting.

### Tissue collection and histological examination

In this part, at least three adult mice for each group were experimented in each group. After euthanasia, testes and caudal epididymides were dissected immediately. The samples were fixed in 4% paraformaldehyde or Bouin’s fixative overnight, dehydrated in an ethanol series, and embedded in paraffin wax. Then, the samples were cut into 5 μm sections with a microtome. After 42 °C overnight drying, the sections were deparaffinized in xylene, hydrated by a graded alcohol series, and stained with hematoxylin and eosin for histological analysis. Images were collected with a Nikon inverted microscope with a charge-coupled device (CCD) (Nikon, Eclipse Ti-S, Tokyo, Japan).

### Immunofluorescence

Testes were fixed in 4% paraformaldehyde (pH 7.4) overnight at 4 °C, dehydrated, and embedded in paraffin. The samples were cut into 5μm sections with a microtome. Then, the sections were deparaffinized, immersed in sodium citrate buffer (pH 6.0) and heated for 15 min in a microwave for antigen retrieval. After blocking with 5% BSA, samples were incubated with primary antibodies at 4 °C overnight. The samples were incubated with an appropriate FITC-conjugated secondary antibody. Finally, nuclei were stained with DAPI. Images were captured by using confocal microscope (Zeiss 880).

For the combination of lectin histochemistry and IHC, sampls were first treated with IHC for cell markers observation and then with lectin (1:400) 30 min at room temperature to visualize the acrosomes.

### TUNEL assay

TUNEL assay was carried out in accordance with the DeadEnd^TM^ Fluorometric TUNEL System (Promega BioSciences, Madison, WI, USA). Images were captured using a laser scanning confocal microscope (Zeiss 880 META).

### Flow cytometry analysis

The cauda epididymides were dissected from 2-month adult mice. Cells were extruded from the cauda epididymides and washed with PBS three times at 37 C. Then, the cells were collected by centrifugation at 600× *g* for 5 min. Next the cells were suspended and stained with Hoechst 33342 for 30 min, and were then analyzed using a FACSCalibur flow cytometer (BD).

### Isolation of mouse primary sertoli cells

Primary Sertoli cells were isolated using a method previously described by van der Wee with minor modification [47]. Briefly, the testes of 5–7 dpp mice were removed and decapsulated under a dissection microscope. The seminiferous tubules were torn into small pieces and washed with PBS three times. The tubules were incubated in PBS containing 2 mg/ml collagenase (Sigma, C5138, St. Louis, MO, USA) and 1 mg/ml DNase I (Solarbio, D8071) at 37 °C for 30 min with gentle shaking. Then, the cells were collected by centrifugation at 100× *g* for 1 min at 4 °C and washed twice with PBS. Next, the cells further digested with 2 mg/ml collagenase I, 1 mg/ml DNase I, and 1 mg/ml hyaluronidase (Sigma, H3506) for 20–30 min at 37 °C with gentle shaking. The tubules were allowed to settle and were then washed twice with PBS before being digested with 2 mg/ml collagenase I, 1 mg/ml DNase I, 2 mg/ml hyaluronidase, and 1 mg/ml trypsin(Sigma, T8003) for 20 min at 37 °C. This final digestion step resulted in a cell suspension containing primarily Sertoli cells and type A spermatogonia. The dispersed cells were then washed twice with DMEM/F12 and placed into culture dishes in DMEM/F12 containing 10% fetal calf serum and incubated at 37 °C and 5% CO_2_. After a 1-day culture, the cells were treated with a hypotonic solution (20 mM Tris, pH7.4) for 2 min to remove germ cells. After 3 days culture, total proteins were extracted as described above for Western blot.

### Phosphoproteome sample preparation and phosphopeptide enrichment

For phosphoproteome preparation, primary Sertoli cells from ten mice in each group were pooled to obtain about 1 mg of protein lysate. Primary Sertoli cells were isolated using a method above described by van der Wee with minor modification [47]. The phosphoproteome experiment was supported by Jingjie PTM BioLabs. The Sertoli cells were lysed with lysis buffer supplemented with Phosphatase Inhibitor Cocktail and Protease Inhibitor Cocktail and precipitated with 4 × volumes of −20 °C acetone for 4 h. Then precipitated protein was collected by centrifuging for 5 min at 4500 g (4 °C), pellets washed twice with −20 °C 80% acetone, and air-dried upside down for ~10 min at RT or until no residual acetone odor remained. Pellets were resuspended in 8 M carbamide. Then protein concentration was determined with a BCA assay kit according to the manufacturer’s instructions. Then samples were digested with trypsin. For phosphopeptide enrichment, digested peptide mixtures were incubated with an IMAC microsphere suspension. The IMAC microspheres with enriched phosphopeptides were collected by centrifugation, and the supernatant was removed. Then the IMAC microspheres were washed. Finally, the supernatant was collected and lyophilized for the LC-MS/MS.

### LC-MS/MS analysis

The peptides were dissolved in 0.1% formic acid, directly loaded onto a home-made reversed-phase analytical column. Peptides were separated and were subjected to Capillary source followed by the tims-TOF Pro (Bruker Daltonics) mass spectrometry.

### Database search

The resulting MS/MS data were processed using MaxQuant search engine (v.1.6.6.0). Tandem mass spectra were searched against the Mus musculus swissprot database (17045 entries) concatenated with reverse decoy database. Trypsin/P was specified as a cleavage enzyme allowing up to two missing cleavages. False discovery rate (FDR) was adjusted to < 1%.

### Phosphoproteome bioinformatics data analysis

We performed bioinformatic analysis by using the Perseus software environment. Statistical analysis of phosphoproteome was performed on logarithmized intensities for those values that were found to be quantified in any experimental condition. Gene Ontology (GO) annotation proteome was derived from the UniProt-GOA database (http://www.ebi.ac.uk/GOA/). Kyoto Encyclopedia of Genes and Genomes (KEGG) database was used to annotate protein pathway.

### Plasmid construction and In vitro phosphatase assay

Mouse *Ppp6c* gene (NM_024209.3) was cloned into pCS2+ vector and mouse *Ctnnb1* gene (NM_001165902) was cloned into pCMV vector. Mutagenesis was used to gain plasmids encoding PPP6C with amino acids Asp84 replaced by Asn according to the manufacturer’s instruction (KOD -Plus- Mutagenesis Kit, SMK-101). 293 T cells were transfected with PPP6C-Myc, PPP6C-D84N-Myc, or CTNNB1-HA plasmid. Then, we performed immunoprecipitations by using anti-Myc or anti-HA antibody, after 36 h of transfection. After several washes with PBS, immunoprecipitated PPP6C-Myc or PPP6C-D84N-Myc and CTNNB1-HA were incubated in phosphatase assay buffer at 25 °C for 1 h for the phosphatase assay. The samples were diluted with 2x loading buffer. After being boiled at 95 °C for 10 min, the samples were used for immunoblotting.

### Statistical analysis

All assays were performed at least three times. Paired two-tailed Student’s t-test was used for statistical analysis. Data were presented as mean ± SEM and *P* < 0.05(*), 0.01(**) or 0.001(***) was considered statistically significant.

## Supplementary information

Supplementary Table 1

Histological examination of the epididymides at different ages.

PPP6c depletion results in the abnormality of sperm.

The morphologic observation and histological examination of the testes at different ages.

Functional enrichment of altered phosphoproteome.

Supplement Figure Legends

## Data Availability

All data, including its supplementary information files, supporting the findings of this study are included in this published article. The mass spectrometry proteomics data have been deposited to the ProteomeXchange Consortium (http://proteomecentral.proteomexchange.org) via the PRIDE partner repository with the dataset identifier PXD028051.
