# Supplementary figures and images for: Specific deletion of protein phosphatase 6 catalytic subunit in Sertoli cells leads to disruption of spermatogenesis
Source: Cell Death Dis. 2021 Sep 27;12(10):883. doi: 10.1038/s41419-021-04172-y (PMC8476514; doi:10.1038/s41419-021-04172-y)

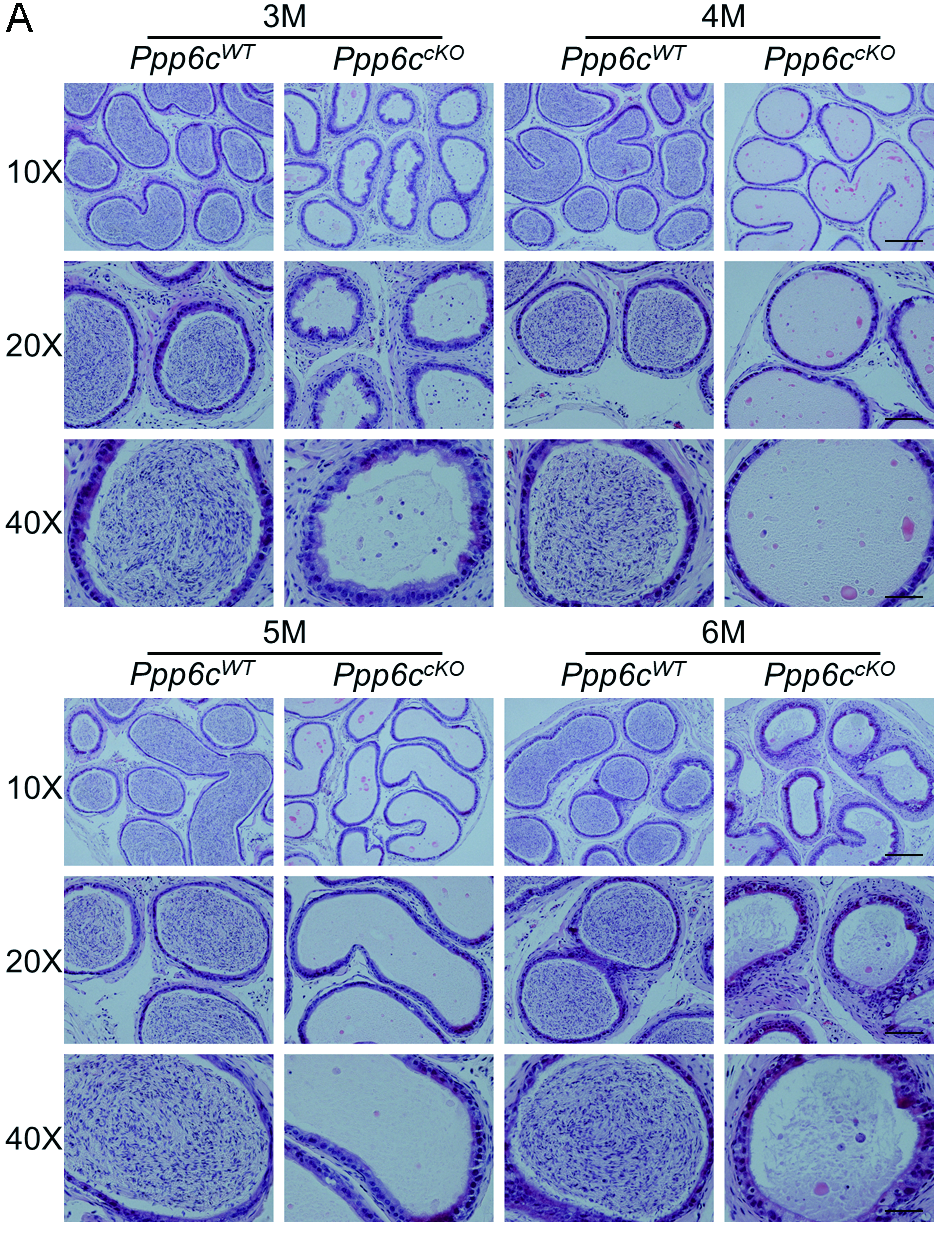

Supplement: Supplementary file 2 — Histological examination of the epididymides at different ages. [file 41419_2021_4172_MOESM2_ESM.tif]

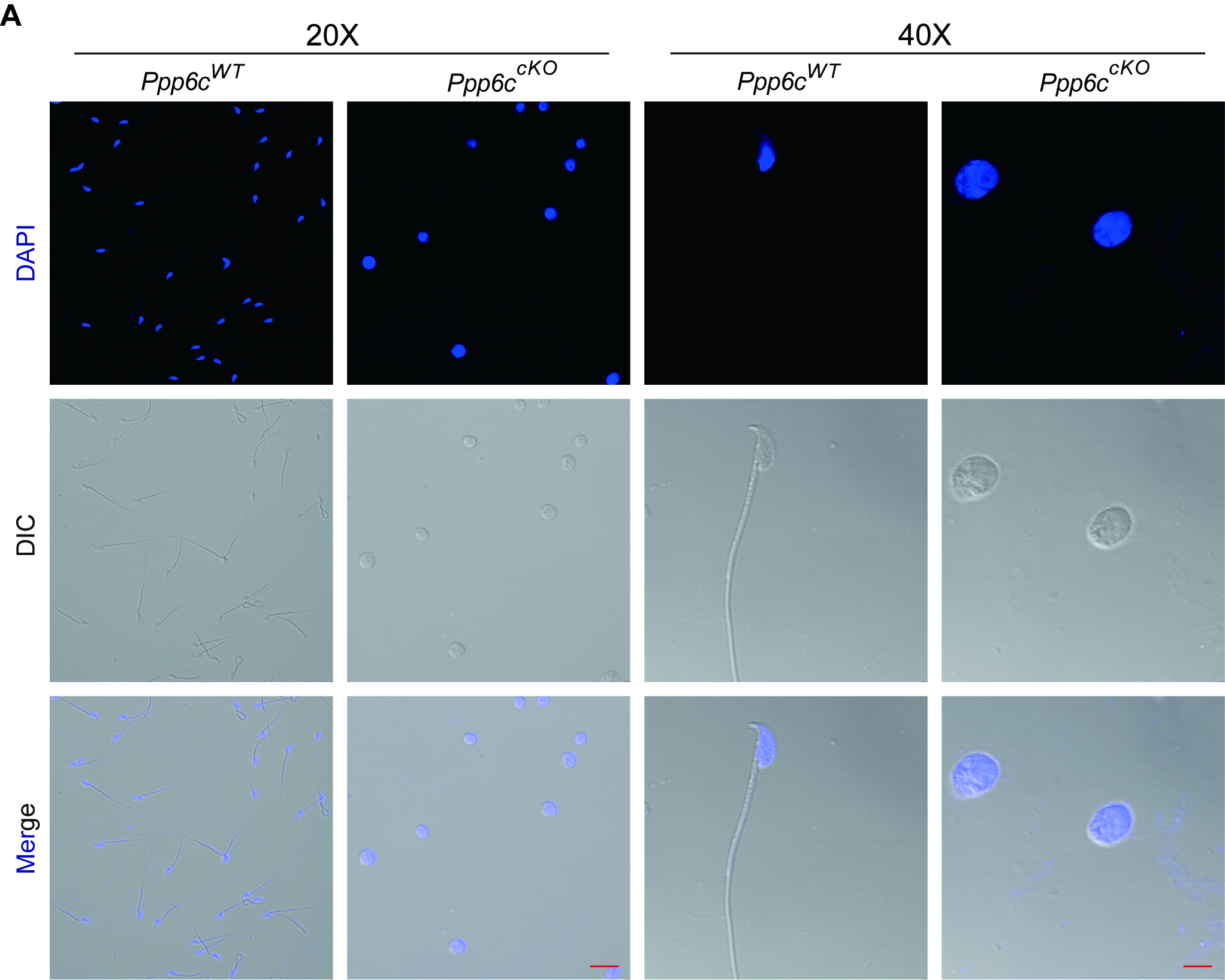

Supplement: Supplementary file 3 — PPP6c depletion results in the abnormality of sperm. [file 41419_2021_4172_MOESM3_ESM.tif]

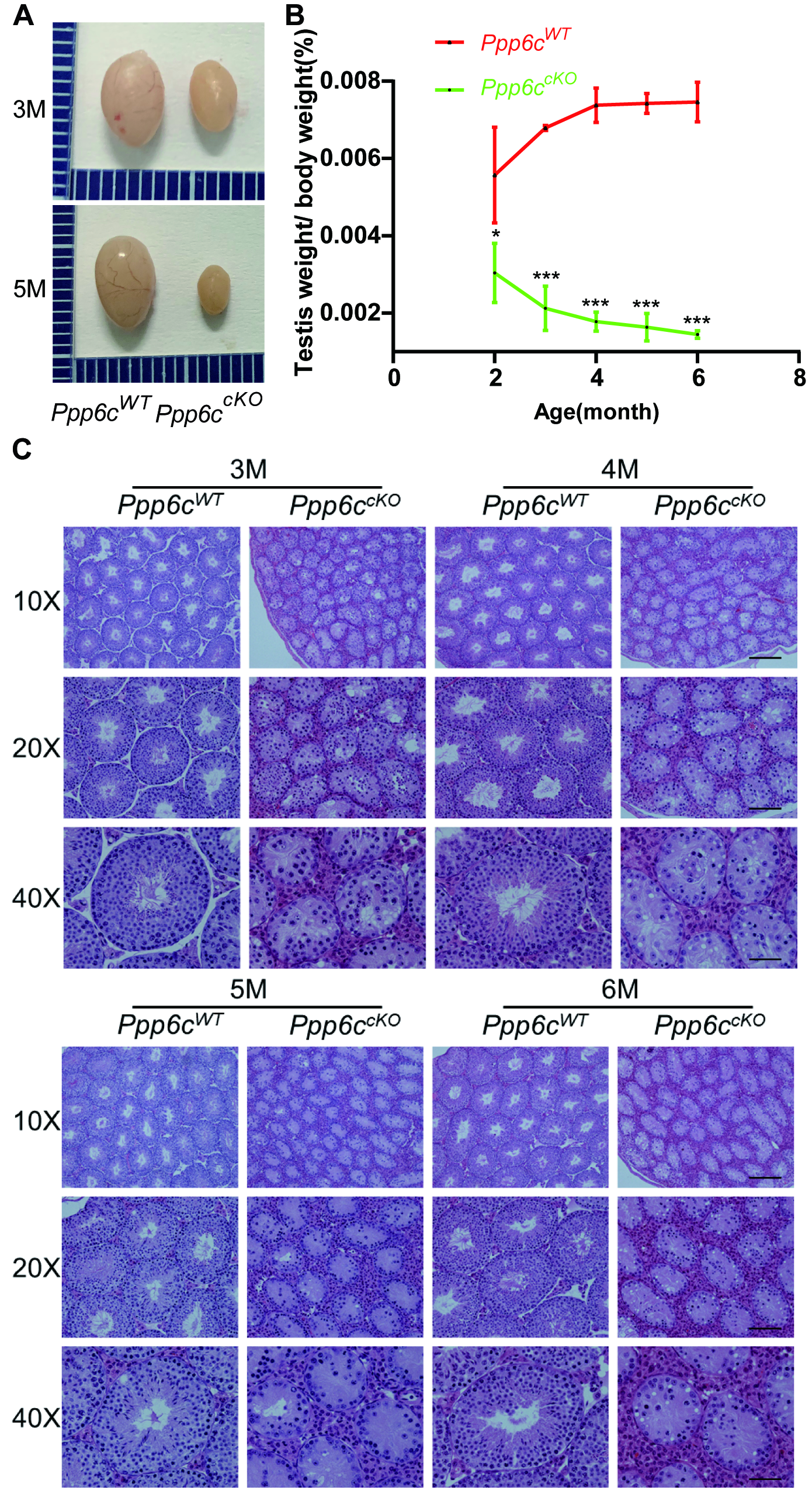

Supplement: Supplementary file 4 — The morphologic observation and histological examination of the testes at different ages. [file 41419_2021_4172_MOESM4_ESM.tif]

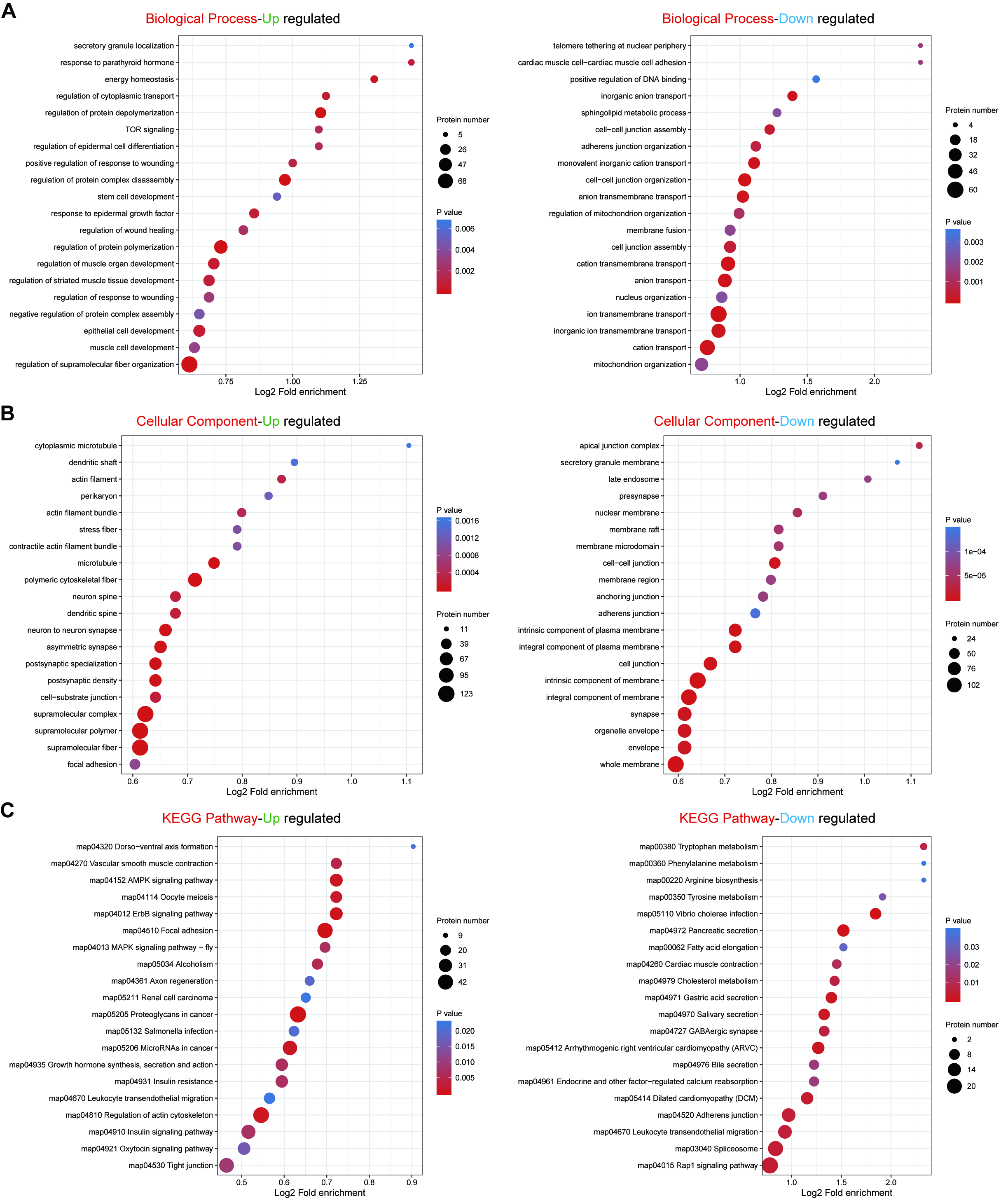

Supplement: Supplementary file 5 — Functional enrichment of altered phosphoproteome. [file 41419_2021_4172_MOESM5_ESM.tif]
