# Supplementary material for: Specific deletion of protein phosphatase 6 catalytic subunit in Sertoli cells leads to disruption of spermatogenesis
Source: Cell Death Dis. 2021 Sep 27;12(10):883. doi: 10.1038/s41419-021-04172-y (PMC8476514; doi:10.1038/s41419-021-04172-y)
Supplement: Supplementary file 6 — Supplement Figure Legends [file 41419_2021_4172_MOESM6_ESM.docx]

**Fig.S1.** **Histological examination of the epididymides at different ages.**

(A) Histological examination of the epididymides of the *Ppp6c^WT^* and *Ppp6c^cKO^* mice in 3 months, 4 months, 5 months and 6 months. Scale bar: (top) 200 μm; (middle) 100 μm; (bottom) 50 μm. At least 3 mice of each genotype were used for analysis.

**Fig.S2. PPP6c depletion results in the abnormality of sperm.**

(A) Single sperm image of the epididymides of the *Ppp6c^WT^* and *Ppp6c^cKO^* mice indicated the morphology of sperm. DAPI (blue). Scale bar: (left) 50 μm; (right) 20 μm. At least 3 mice of each genotype were used for analysis.

**Fig.S3. The morphologic observation and histological examination of the testes at different ages.**

(A) The morphologic observation of testes of the *Ppp6c^WT^* and *Ppp6c^cKO^* mice in 3 months and 5 months. At least 3 mice of each genotype were used for analysis. (B) Testis weight to body weight ratio of *Ppp6c^WT^* and *Ppp6c^cKO^* mice in 2months, 3 months, 4 months, 5 months and 6 months. Data are presented as the mean ± SEM. P<0.05(*), 0.01(**) or 0.001(***). (C) Histological examination of the seminiferous tubules of the *Ppp6c^WT^* and *Ppp6c^cKO^* mice in 3 months, 4 months, 5 months and 6 months. Scale bar: (top) 200 μm; (middle) 100 μm; (bottom) 50 μm.

**Fig.S4 Functional enrichment of altered phosphoproteome.**

(A) The GO-based enrichment analysis of biological processes. (p-value < 0.05). (B) The GO-based enrichment analysis of cellular compartment. (p-value < 0.05). (C) Enrichment of pathway analysis. (p-value < 0.05).

**Supplementary Table 1. The detailed information of differential proteins and phosphorylation sites.**

This table contains all detailed information of differential proteins and phosphorylation sites in this study.
